# An agent-based nested model integrating within-host and between-host mechanisms to predict an epidemic

**DOI:** 10.1371/journal.pone.0295954

**Published:** 2023-12-15

**Authors:** Yuichi Tatsukawa, Md. Rajib Arefin, Kazuki Kuga, Jun Tanimoto

**Affiliations:** 1 Interdisciplinary Graduate School of Engineering Sciences, Kyushu University, Fukuoka, Japan; 2 MRI Research Associates Inc., Tokyo, Japan; 3 Department of Mathematics, University of Dhaka, Dhaka, Bangladesh; 4 Faculty of Engineering Sciences, Kyushu University, Fukuoka, Japan; Universidad Rey Juan Carlos, SPAIN

## Abstract

The COVID-19 pandemic has remarkably heightened concerns regarding the prediction of communicable disease spread. This study introduces an innovative agent-based modeling approach. In this model, the quantification of human-to-human transmission aligns with the dynamic variations in the viral load within an individual, termed “within-host” and adheres to the susceptible–infected–recovered (SIR) process, referred to as “between-host.” Variations in the viral load over time affect the infectivity between individual agents. This model diverges from the traditional SIR model, which employs a constant transmission probability, by incorporating a dynamic, time-dependent transmission probability influenced by the viral load in a host agent. The proposed model retains the time-integrated transmission probability characteristic of the conventional SIR model. As observed in this model, the overall epidemic size remains consistent with the predictions of the standard SIR model. Nonetheless, compared to predictions based on the classical SIR process, notable differences existed in the peak number of the infected individuals and the timing of this peak. These nontrivial differences are induced by the direct correlation between the time-evolving transmission probability and the viral load within a host agent. The developed model can inform targeted intervention strategies and public health policies by providing detailed insights into disease spread dynamics, crucial for effectively managing epidemics.

## Introduction

The COVID-19 pandemic presented challenges in forecasting the dynamics of communicable diseases. The seminal work of Kermack & McKendrick in 1927 marked the inception of quantitative predictions in epidemic spread [1], which was followed by numerous groundbreaking studies. In epidemiological research, COVID-19 has garnered widespread attention from both the scientific community and the general public. Media coverage has popularized the concept of the “basic reproduction number,” denoted as *R*_0_. The basic reproduction number *R*_0_ represents the average number of the secondary infections generated by a primary infection in a fully susceptible population during the infectious period [2–4]. It is a critical statistical index for indirectly evaluating the infectiousness of a communicable disease.

**Fig 1:**
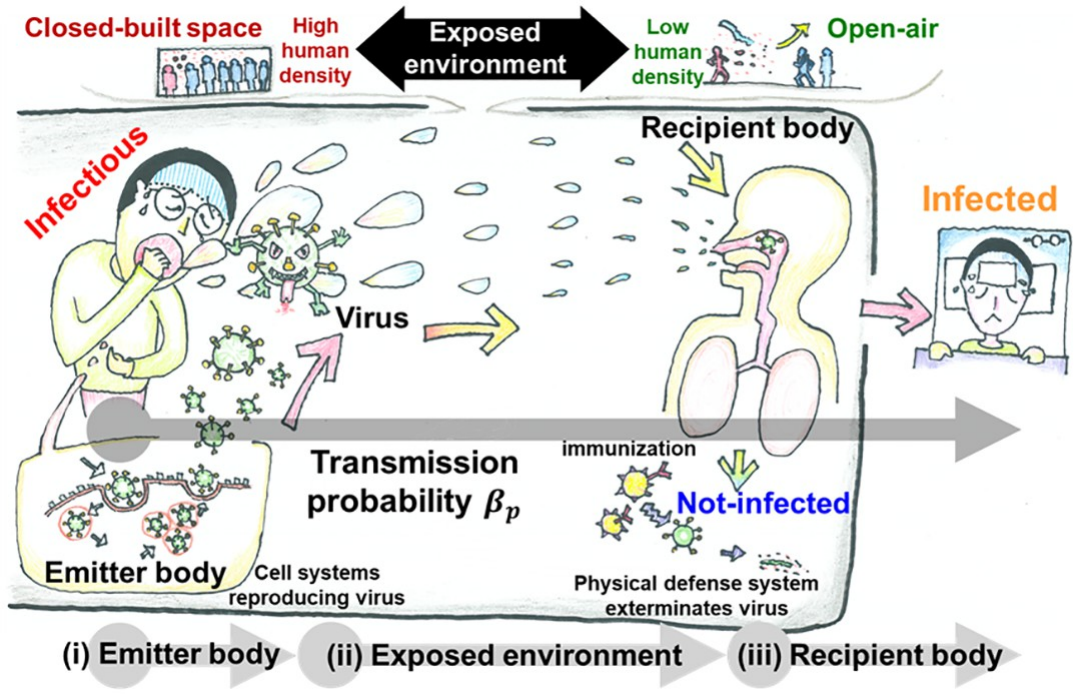
Schematic representation of transmission probability. Entire transmission process is threefold: (i) emitter body, (ii) exposed environment, and (iii) recipient body. The infectious emitter releases the virus in the environment—either indoor or outdoor. Compared to the opposite scenario, an indoor and densely populated environment is a favorable condition for virus transmission. The recipient absorbs the virus from the environment and may become infected. These three factors may combinedly determine the transmission probability.

As established in existing research, infectious diseases transmit through physical contact [2,5,6]. A practical approach to modeling epidemic spread involves the utilization of complex network topology, where nodes symbolize individuals and links denote the interactions [5,7]. In this framework, following the SIR process, an infected individual transmits the disease to a susceptible neighbor with a transmission probability *β*_*p*_. Fig 1 schematically explains the concept of transmission probability *β*_*p*_, which comprises three components: (i) emitter body, (ii) exposed environment, and (iii) recipient body. The emitter body pertains to the viral load in an infectious agent, where an increase in virus production raises *β*_*p*_. The exposed environment—varying from indoor to open-air and from densely to less densely populated areas—also significantly influences the trend of *β*_*p*._

Regarding the third component, the recipient body, the likelihood of infection relies on the amount of virus inhaled from the emitter and the activity level of the biological defense system of the recipient. These factors collectively determine a composite probability for *β*_*p*_. However, the time scales for these components vary significantly. The overall time scale for *β*_*p*_ is expected to be on the order of days, similar to the time frame for processes within the emitter’s body. In contrast, the time scales for the exposed environment and recipient body are considerably shorter, typically in minutes or seconds. The instantaneous turbulent effects caused by environmental factors and agent movement poses challenges for accurate quantification. Owing to this disparity in time scales, incorporating all three influences on *β*_*p*_ is complex. Nevertheless, if the latter two components are overlooked owing to their brief occurrence, *β*_*p*_ estimation primarily considers the contribution of the emitter body. Therefore, the transmission probability can be modeled by observing the viral load (represented as (*t*)) in a human body, resulting in a formulation of *β*_*p*_ as a function of time *t* and viral load *V*(*t*), expressed as *β*_*p*_(*t*, *V*(*t*)). This study explores whether the time- and viral load-dependent transmissibility influences the epidemic outcomes compared to traditional models.

Most conventional SIR models operate on the assumption of a constant transmission rate, especially when employing the ordinary differential equations (ODE) approach, as reported in references [3,8,9], or a constant transmission probability in the context of multi-agent simulations (MAS), as indicated in [7,10–12]. Several studies including [13–16] have explored time-dependent transmission rates or probabilities, especially in networked models. These studies primarily focus on the impact of various interventions, such as mask usage [17,18], social distancing measures [19–21], and reduction of social activities, exemplified by the implementation of lockdowns [22]. These interventions are often not in place initially but are introduced after a specific period.

In the current study, a novel agent-based framework for epidemic spread is proposed, incorporating viral kinetics within the human body. This model is categorized into two components: the “within-host” mechanism, representing viral kinetics, and the “between-host” aspect, quantifying the dynamics of disease spread through human-to-human interactions within a network. Consequently, the within-host mechanism is integrated into the agent-based between-host dynamics, governed by the SIR process. In this model, the transmission probability during the infectious period is determined by the viral load in each infected agent.

The nested models have garnered significant interest in recent years. For an in-depth review, readers may consult the article by Martcheva et al. [23]. This research provides a concise overview of relevant studies. Pereira et al. [24] developed a nested model linking within-host dynamics to a time-discretized between-host dynamics, specifically tailored for tuberculosis, thereby limiting its application to general SIR-type dynamics. Feng et al. [25] employed a mean-field approach to integrate within-host viral dynamics with a susceptible–infected (SI) epidemic model for HIV. Steinmeyer et al. [26] established a model featuring viral load dynamics (within-host), which connects to specific between-host dynamics by estimating the viral emission and absorption between emitters and recipients. However, their model does not incorporate host-contact network structures, a critical component in epidemic modeling, as the degree of connectivity among individuals significantly influences disease spread [26]. For example, epidemic propagation in a scale-free network [27] tends to be hierarchical, initially impacting highly connected individuals before reaching those with fewer connections [26,28,29]. A related methodology is detailed in Ref. [30].

The novelty of our model lies in its integration of within-host viral dynamics with a network-restricted (scale-free) SIR epidemic model. This model establishes a power-law relationship between transmission probability and the viral load of an infected agent. The present focus is on examining the final epidemic size, peak number of infections, and the timing of these peaks under this framework and comparing these outcomes with those predicted by conventional models.

This study is structured as follows: In the Methods section, the nested model is described, combining the ODE-based within-host viral dynamics with agent-based between-host disease spreading dynamics. The Results section presents findings from numerical experiments pertaining to the nested model. These results are thoroughly analyzed in the subsequent Discussion section. Finally, the conclusions of this study are summarized at the end.

## Methods

**Fig 2:**
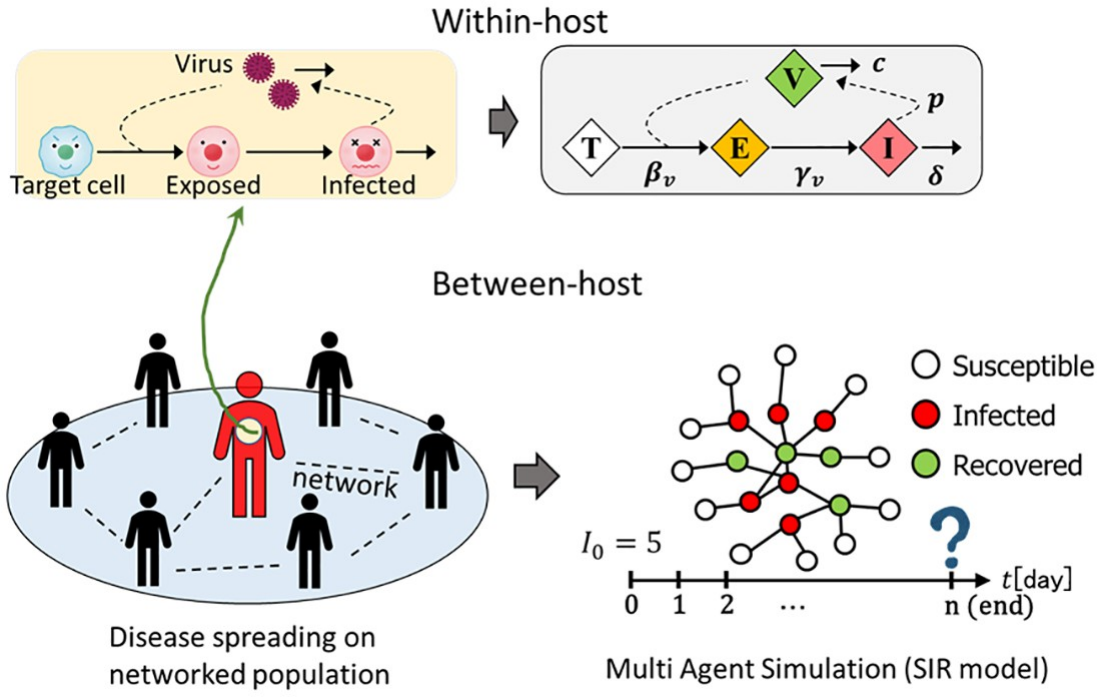
A schematic explanation of the nested model. Viral kinetics inside the host body is formulated by a deterministic ordinary differential equation-type target cell-exposed cell-infected-viral load process. The between-host part, following the SIR process, is implemented by an agent-based stochastic simulation model on a network. The simulation starts with randomly assigning five initially infected agents on the network. Each infected agent may infect susceptible neighbors with a time-varying transmission probability *β*_*p*_(*t*), which depends on the viral load inside the host.

Fig 2 provides a schematic depiction of the current model. The within-host component, structured as an ODE-type target cell-exposed-infected-viral load (TEIV) process, quantifies viral dynamics in each agent. Conversely, the between-host aspect, modeled using the MAS approach and adhering to the SIR process, chronicles sequential stochastic events on a complex network. The study employs either a complete graph, representing a well-mixed situation, or a Barabási–Albert scale-free graph (BA-SF) [27] with a population size of *N* = 10^4^. In the BA-SF network, a small number of nodes, known as hubs, have significantly higher degrees than other nodes. Specifically, we assume a BA-SF network with an average degree of 8 (⟨𝑘⟩ = 8). The framework includes two temporal dimensions. Within each host agent, the infection age denotes the period during which the viral load fluctuates. This duration ceases when the host is no longer infectious, marked by a substantially reduced viral load. In the between-host context, the focus shifts to monitoring various agents classified as susceptible, infected with a specific infection age, or recovered. The infection age correlates with a particular viral load, influencing the likelihood of infecting susceptible neighbors. The process concludes when no infected agents remain. To derive the average values for each quantity, we conducted 10^3^ realizations, each commencing with five randomly positioned infected hosts.

### Within-host model

**Fig 3:**
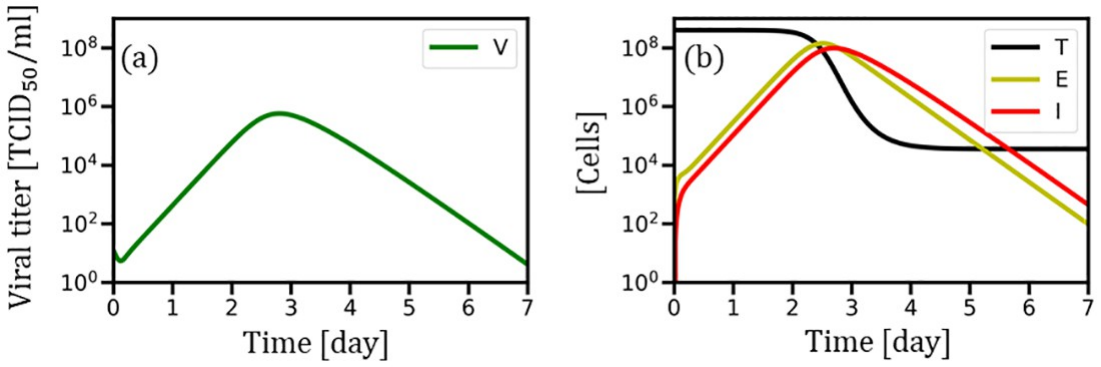
Deterministic dynamics of the within-host mechanism. (a) temporal evolution of the viral load; (b) time series of the number of target cells, exposed cells, and infected cells.

In this study, we adopt the TEIV process outlined by Baccam et al. [31], which was empirically determined using subjects voluntarily infected with influenza A. The formulation of this process is as follows:

$$\begin{array}{l} \frac{dT\left(t\right)}{dt}=-\beta_{v}T\left(t\right)V\left(t\right),\\ \begin{array}{l} \frac{dE\left(t\right)}{dt}=\beta_{v}T\left(t\right)V\left(t\right)-\gamma_{v}E\left(t\right),\\ \frac{dI\left(t\right)}{dt}=\gamma_{v}E\left(t\right)-\delta I\left(t\right),\\ \frac{dV\left(t\right)}{dt}=pI\left(t\right)-cV\left(t\right), \end{array} \end{array}$$ (1)

where *T*(*t*), 𝐸(*t*), 𝐼(*t*), and *V*(*t*) represent the number of target, exposed, and infected cells and viral load, respectively, at time *t*. Experimentally identified parameters and initial conditions are stated as follows [31]: infection rate, *β*_*V*_ = 1.466 × 10^−5^) [(TCID_50_/mL)^−1^ day^−1^]; transition rate, *γ*_*V*_ = 3.274 [day^−1^]; production rate, *p* = 5.826 × 10^−2^ [(TCID_50_/mL)^−1^ day^−1^]; cell death rate, *δ* = 3.934 [day^−1^]; virus extraction rate, *c* = 9.575 [day^−1^]; *T*(0) = 4 × 10^8^ [cells]; and *V*(0) = 12.41 [TCID_50_/mL]. Note that the median tissue culture infectious dose is abbreviated as TCID_50_ and defined as the dilution of a virus required to infect 50% of a given cell culture [32]. The outcomes depicted in Fig 3(a) and 3(b) were obtained using Eq (1). Consequently, the viral load (panel (a)) was deterministically quantified for a duration of seven days following the infection of a focal agent.

### Disease spreading process: Between-host model

For the SIR process, an agent-based model was constructed. Each agent is situated on a node of the underlying network, either a complete graph or a BA-SF network, with links between nodes signifying physical contact. At the start of a simulation episode, all agents are classified as susceptible, except for a few initially infected agents (herein, *I*_0_ = 5), randomly distributed within the network. In this agent-based simulation, the transition from susceptible (S) to infected (I) status is considered stochastic.


$$I+S\overset{\beta_{p}}{\to }I+I,$$ (2)

In this model, if an infectious agent has a susceptible neighbor, there exists a probability, denoted as *β*_*p*_, that the neighbor will become infected Eq (2). We assume that each infected agent, on average, recovers after a period of seven days. Therefore, the reciprocal of the recovery rate, *γ*^−1^ = 7 days, i.e., *γ* = 1/7 given that the recovery time follows an exponential distribution. This infected period is referred to as *T*_*inf*_ (= 7 days). Referring to the typical basic reproduction number for seasonal influenza, we set *R*_0_ = 2.5. Consequently, the transmission probability (per link) in the MAS corresponding to *R*_0_ is expressed as follows:

$$\beta_{p}=\frac{R_{0}\gamma }{\left\langle k\right\rangle }.$$ (3)

For comparative analysis, we also utilize a conventional SIR model adapted to the MAS approach. This model employs a time-constant transmission probability throughout the infectious period, here defined as seven days. Practically, for the complete graph, we assumed *$\beta_{p}=\frac{2.5∙1/7}{10^{4}-1}=3.57\times 10^{-5}$* for the BA-SF network, $\beta_{p}=\frac{2.5∙1/7}{8}=4.46\times 10^{-2}$.

### Bridging between within-host and between-host

**Fig 4:**
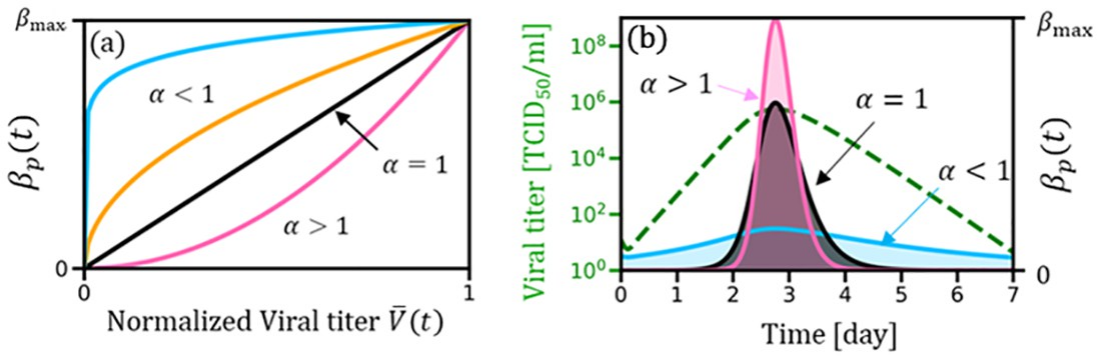
(a) Power-law relationship between transmission probability *β*_*p*_(*t*) and normalized viral titer $\bar{V}\left(t\right)$ depicted by the relationship given in Eqs (4) and (5). Parameter *α* > 1(*α* < 1) renders the curve convex (concave). System ranges from $\left(\bar{V},\beta_{p}\right)=\left(0,0\right)$ up to (1, *β*_*max*_) with the monotonically increasing tendencies depending on *α*. (b) viral load *V*(*t*) (green dashed line) and the transmission probability *β*_*p*_(*t*) for various selection of *α*, resulting from Eq (4). Here, we selected *β*_max_ = 0.3 and *γ* = 1/7 to generate panel (b). Note that the total area under each curve in panel (b) is the same and equal to that of the time-constant transmission probability. Note that panel (b) yields information about the generation time distribution, i.e., the time interval between infections in infector-infected pairs.

The present model illustrates the dependence of the transmission probability of an agent *i*, denoted by $\beta_{p}^{i}\left(t\right)$, on its viral load *V*(*t*) (as in Eq (1)). However, the exact kinetic relationship between viral load and transmission probability for epidemics like seasonal influenza or COVID-19 remains largely undefined [33]. Handel et al.[34] comprehensively reviewed this issue. Park et al. [35] endeavored to estimate this relationship using experimental data derived from horses rather than humans. Saad-Roy et al. [36] suggested that the transmission rate of influenza roughly correlates with the power of the viral load. Extensive data analysis by He et al.[37] on the COVID-19, Larremore et al.[38] to hypothesize three potential functional relationships between the viral load and transmission rate: (i) *β*(*t*) is proportional to log[*V*(*t*)], (ii) *β*(*t*) is linearly proportional to *V*(*t*), and (iii) *β*(*t*) is saturated with a certain fixed value if *V*(*t*) exceeds a threshold. Note that time *t* refers to the age of infection inside the host. Considering these studies, we postulated a power-law relationship between the transmission probability and viral load as follows:

$$\beta_{p}^{i}\left(t\right)=\beta_{max}{\bar{V}}_{i}{\left(t\right)}^{\alpha },$$ (4)

$${\bar{V}}_{i}\left(t\right)=V_{i}\left(t\right)/V_{max},$$ (5)

where *V*_*i*_(*t*) denotes the viral load of agent *i* at time *t*, obeying to *V*(*t*) in Eq (1), and *V*_*max*_ indicates its peak value in the time evolution, as depicted in Fig 3(a). *V*_*i*_(*t*) in Eq (5) depicts the normalized virus level of agent *i*. *β*_*max*_ and *α* are considered positive parameters. Fig 4(a) displays that *α* controls the power-law tendency. In particular, *α* = 1 recovers the linear relationship.

The parameter set (*β*_*max*_, *α*) is uniquely determined to satisfy $\int_{t=0}^{T_{inf}}\beta_{p}^{i}\left(t\right)dt=\int_{t=0}^{T_{inf}}\beta_{p}\;dt$, i.e., the area underneath each curve, corresponding to various choices of *α*, is equal and consistent with the case for the time-constant transmission probability (Fig 4(b)). This specific condition is one of the most vital assumptions in the current framework, physically indicating that the time integral of the infectiousness is preserved, regardless of the conventional SIR model (a time constant *β*_p_ by Eq (3)) or the present model (time variable $\beta_{p}^{i}\left(t\right)$ depending on *V*_*i*_(*t*). Based on this assumption, whenever either *β*_*max*_ or *α* is considered a constant control parameter, the remaining one (i.e., either *α* or *β*_*max*_) will be automatically adjusted. Note that as *α* → 0, *β*_*max*_ approximates to a time constant *β*_p_ (by Eq (3)) that recovers the conventional SIR model. Fig 4(b) further elucidates how disease transmission speed varies with different α values. For instance, a curve for α > 1 (pink curve) features a higher peak, indicating a concentration of probability distribution around the mean, which translates to an increased likelihood of transmission between the second and fourth days of the infection period. Conversely, α < 1 corresponds to a lower peak curve (blue curve), suggesting a reduced transmission probability during the infectivity period.

This observation is intricately connected to the concept of generation time [39,40], which calculates the interval between infections in infector–infected pairs. Therefore, the model implies that the generation time for α > 1 is shorter compared to α < 1, indicating faster transmissions in the former scenario. In both cases, the average generation time is estimated to be slightly less than three days. Thus, Fig 4(b) offers insight into the generation time distribution of our model.

## Results

**Fig 5:**
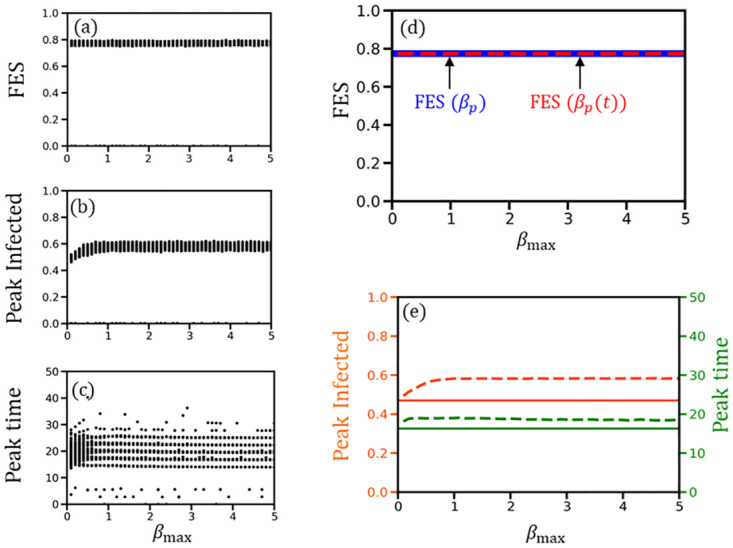
(a), (b), and (c) respectively display all 10^3^ realizations for final epidemic size (FES), peak infected, and its appearing time for the nested model (on Barabási–Albert scale-free network) as a function of *β*_*max*_. (d) Ensemble average of FES (red-dashed line derived from panel (a)) along *β*_*max*_, which is compared with the result obtained from the conventional SIR model (horizontal blue solid line). The assembled averages of the peak infected (orange solid line derived from panel (b)) and the peak time (green solid line derived from panel (c)) are finally compared with the conventional model (solid lines) in panel (e).

Fig 5 presents outcomes for scenarios involving BA-SF networks. Our primary focus is on the final epidemic size (FES), determined by the count of recovered individuals at equilibrium (the point when no infected individuals remain) for each simulation episode. Additionally, we evaluate the peak number of infected individuals and the timing of this peak relative to *β*_*max*_, with values of *β*_*max*_ ranging from 0 to 5.0. Panels (a), (b), and (c) in Fig 5 illustrate all 10^3^ realizations for FES, peak infected, and peak time, respectively. Panel (d) compares the ensemble average of FES in our model to that in the conventional SIR model (depicted as a blue solid line) in a BA-SF network. Panel (e) contrasts the average peak infected number in the nested model (dashed lines) against the conventional model (solid lines).

**Fig 6:**
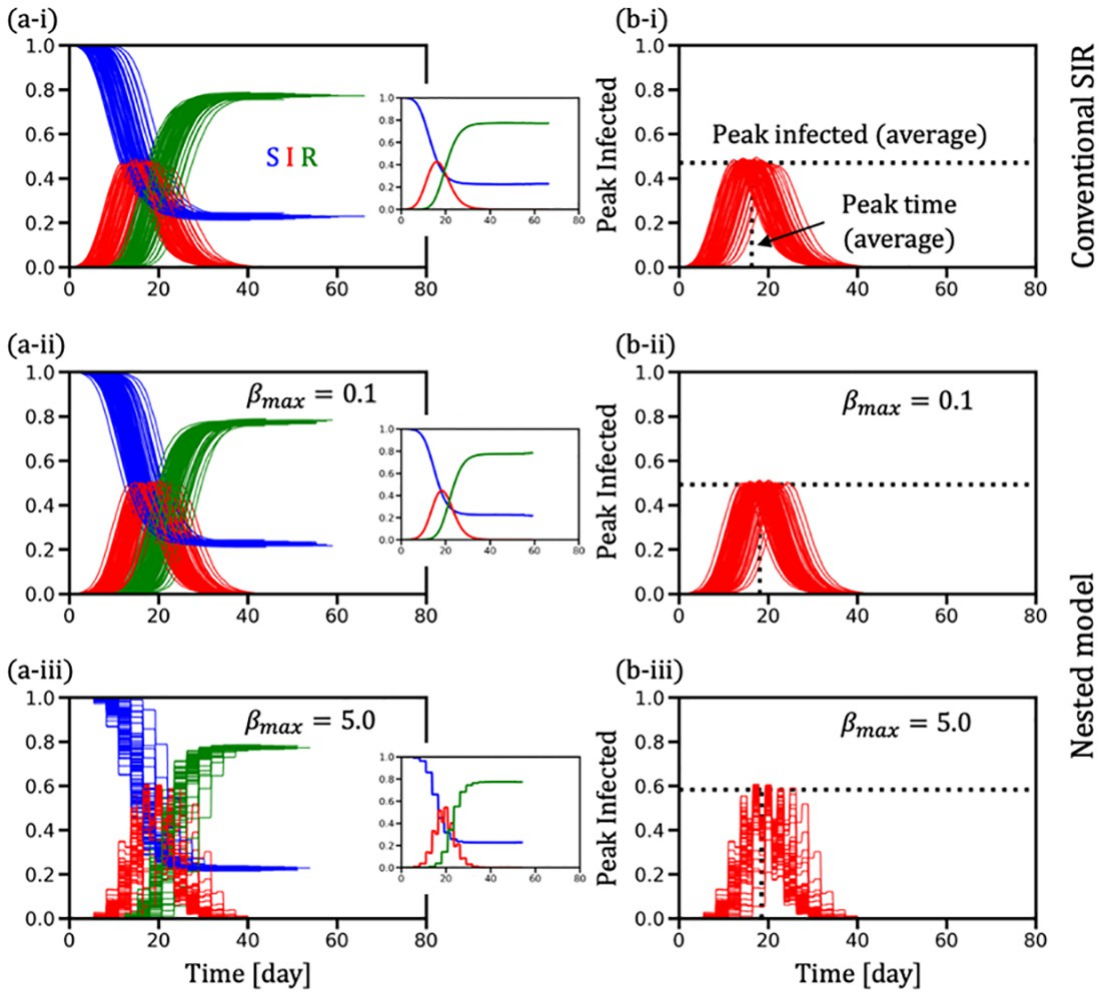
Temporal evolution of the fractions S, I, and R for 10^3^ realizations on the Barabási–Albert scale-free network. (a-*) time series of S, I, and R; (b-*) fraction of infection (I) to illustrate the average peak infected and the average peak time. Insets in panel (a-*) display the average over 10^3^ realizations for the fractions S, I, and R. Panels (*-i) exhibit the case of the conventional SIR model, whereas panels (*-ii) and (*-iii) demonstrate the instances of the nested model corresponding to a lower (*β*_*max*_ = 0.1) and higher (*β*_*max*_ = 5.0)*β*_*max*_.

Fig 6 displays the time-series data for susceptible (S), infected (I), and recovered (R) populations across 10^3^ realizations on the BA-SF network, as observed in panels (a-*). The time series for the fraction of infected individuals is also plotted to highlight the average peak infected level and average peak time in panels (b-*). The top row illustrates results from the conventional SIR model (panels (*-i)), whereas the middle and bottom rows exhibit outcomes from the nested model for lower (*β*_*max*_ = 0.1) and higher (*β*_*max*_ = 5.0) *β*_*max*_ values, respectively. Notably, results in panels (b-ii) and (b-iii) indicate that a higher β_max_ results in a shorter epidemic duration and a more pronounced infection peak. Corresponding findings for a well-mixed setting, which exhibit similar trends (S1 Fig).

## Discussion

A key observation of the nested model is that it yields the same FES as the conventional SIR model (Fig 5(d)). However, marked differences emerge concerning the peak level and its timing (refer to Fig 5(e)) between the nested (dashed lines) and conventional (solid lines) models. Simulations indicate that both the peak infected level and its occurrence time are higher in the nested model compared to the conventional SIR model. This pattern is evident in the well-mixed population scenario on a complete graph (S2 Fig). Notably, a larger *β*_*max*_ produces a more pronounced peak of $\beta_{p}^{i}\left(t\right)$, although with a shorter active duration, characterized by a higher peak and narrower curve width (illustrated by a red line in Fig 4(b)). This result signifies that considering a time-varying transmission probability results in an earlier and higher peak infected level compared to predictions by the conventional SIR model, especially when the characteristic time-variable transmission probability features a narrow and high peak.

As observed from Fig 6, a consistent trend regarding the infected peak is observed in both BA-SF and complete graph scenarios. Nonetheless, the peak times in the nested model are delayed relative to the conventional model, attributable to the heterogeneity effect of the BA-SF compared to the complete graph (as depicted by comparing panel (b-i) with panels (b-ii) and (b-iii) in Fig 6). To validate the robustness of our findings, the simulations were extended to other network types such as small-world [41], random regular, and Erdős–Rényi (ER) random networks [42,43], (S3 Fig). Additionally, various initial conditions were examined (S4 Fig). In each scenario, the FES in both nested and time-constant epidemic models were consistent. Therefore, the phenomenon of identical FES in both conventional and nested models can be considered general, based on the assumption that the time-integrated transmission probability is similar in both frameworks.

In our analysis thus far, we assumed a constant *β*_*max*_ for each agent. However, let us consider a scenario where *β*_*max*_ varies among agents. In this context, we explore the dynamics wherein, during each infection event (i.e., virus transfer) on a scale-free network, the recipient’s *β*_*max*_ is equal to the *β*_*max*_ of the emitter, adjusted by a mutation bias, expressed as $\beta_{max}^{recipient}=\beta_{max}^{emitter}\pm \Delta$, where Δ ∈ [−0.3, 0.3] represents a mutation bias following a uniform distribution.

Under this framework, our simulations demonstrate that the overall *β*_*max*_ value in the population, starting from an initial value (e.g., 1.0 for each of the initially infected agents; *I*_0_ = 5), fluctuates in both upward and downward directions over time. However, during a prolonged epidemic period, *β*_*max*_ tends to settle at a lower value (S5 Fig). We observed that this variable *β*_*max*_ scenario does not significantly impact the FES, peak infected level, or peak time, in comparison to the fixed *β*_*max*_ scenario (Fig 5).

## Conclusions

In this research, we developed a nested within- and between-host model where a global SIR process is driven by an agent-based model on a complex network. This model features a time-varying transmission probability, dependent on the viral load of each infected agent, as determined by the viral kinetics (TEIV process) within the host.

When the time-integrated infectiousness of an infected individual aligns with the conventional framework (i.e., time-constant transmissibility), the FES remains consistent between the nested model and the standard SIR process. However, the nested model exhibits a higher peak infected level, which appears later than that predicted by the conventional SIR model. This deviation results from the time-varying transmission probability characterized by a higher peak and shorter active period, particularly notable in networks with heterogeneous degree distributions. This observation remains unaltered under various initial simulation conditions (S4 Fig). The robustness of the model was further validated across different network topologies, including small-world, random regular, and ER random networks (S3 Fig). Thus, the findings from this model can be regarded as general, provided the baseline assumption of consistent time-integrated transmission probability with the time-constant probability during the infection period is maintained.

The primary limitation of this study includes its assumption of uniform within-host dynamics, potentially oversimplifying the diversity of real-world scenarios. Future research could address this by incorporating more varied within-host dynamics and exploring alternate disease models. Despite this, the research significantly contributes a novel framework bridging individual viral kinetics with epidemic spread, providing practical implications for public health policy and intervention strategies.

## Supporting information

S1 Fig Comparison between the nested and conventional SIR models for the well-mixed setting. (TIF)

S2 Fig Illustrations of final epidemic size, peak infection, and time to attain the peak infection for the well-mixed setting. (TIF)

S3 Fig Final epidemic size and the peak infection time on three different networks. (TIF)

S4 Fig Final epidemic size versus *β*_*max*_ graph on the scale-free network. (TIF)

S5 Fig Evolution of *β*_*max*_ in the time direction. (TIF)

S1 File (CPP)

S2 File (H)

S3 File (CPP)

S4 File (H)

S5 File (CPP)

S6 File (H)

S7 File (CPP)

S8 File (H)

S9 File (H)

S10 File (CPP)

S11 File (H)

S12 File (CPP)

S13 File (CPP)

S14 File (H)

S15 File (CPP)

S16 File (H)
