# Supplementary figures and images for: An agent-based nested model integrating within-host and between-host mechanisms to predict an epidemic
Source: PLoS One. 2023 Dec 15;18(12):e0295954. doi: 10.1371/journal.pone.0295954 (PMC10723725; doi:10.1371/journal.pone.0295954)

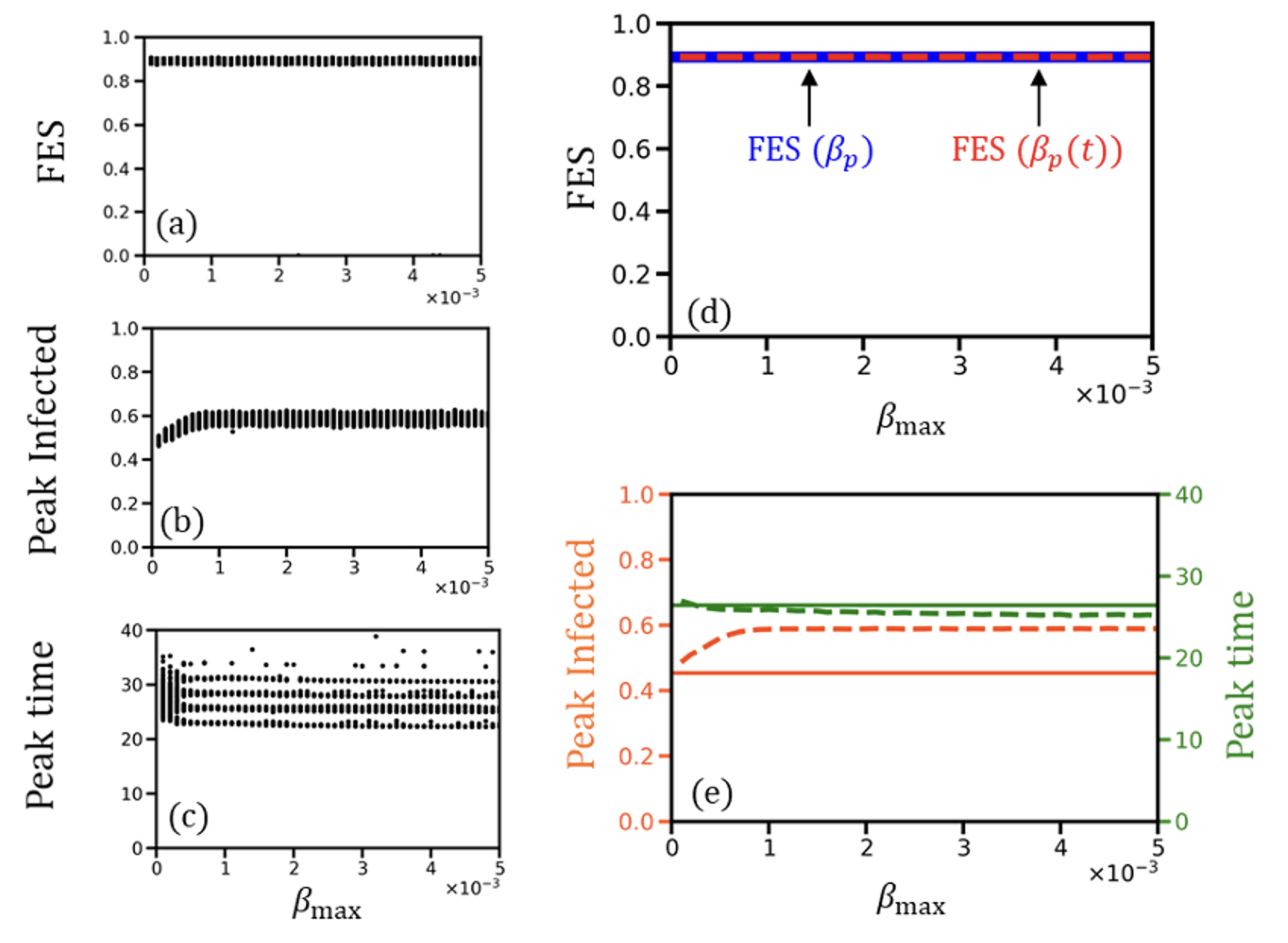

Supplement: S1 Fig — (TIF) [file pone.0295954.s001.tif]

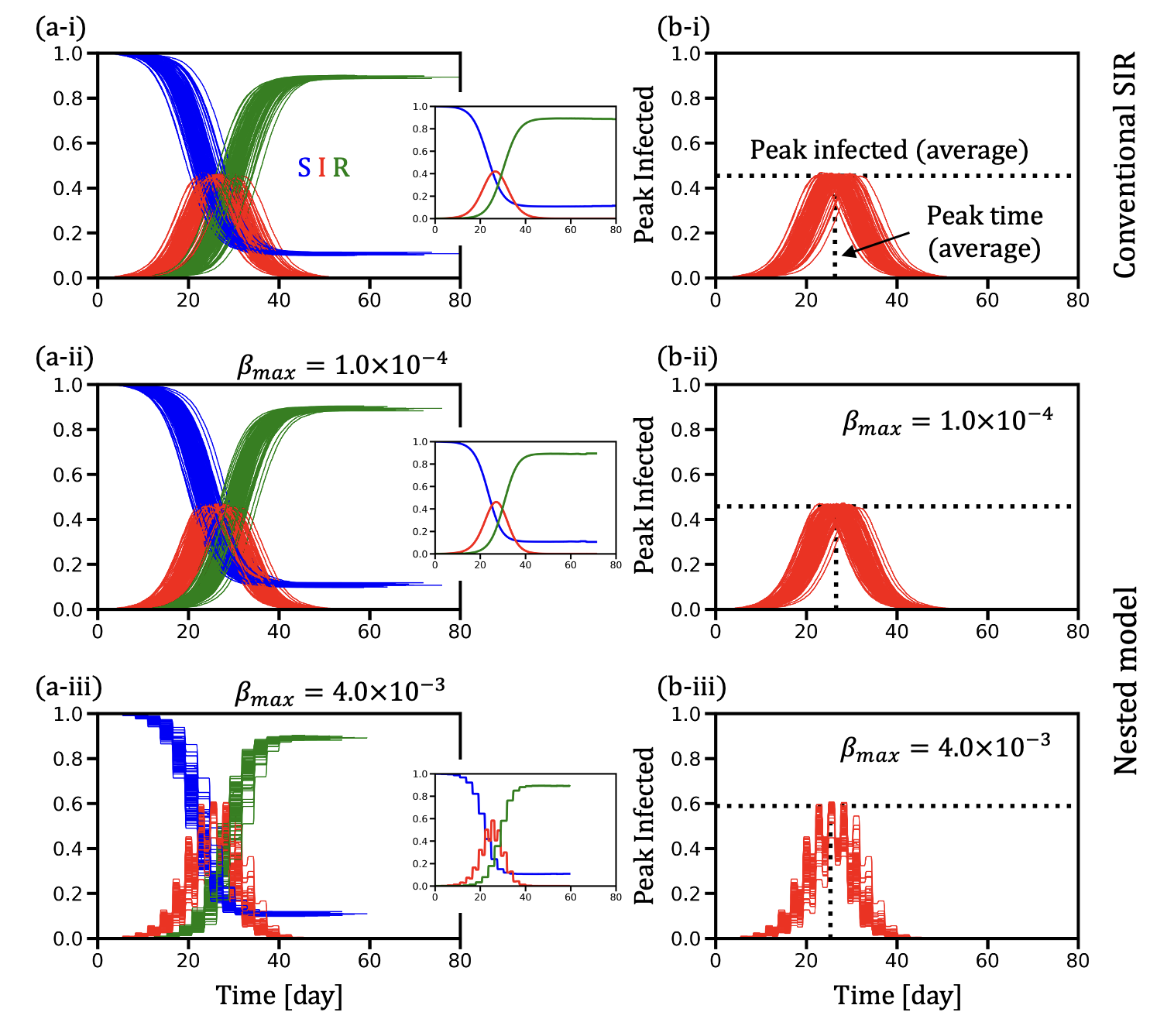

Supplement: S2 Fig — (TIF) [file pone.0295954.s002.tif]

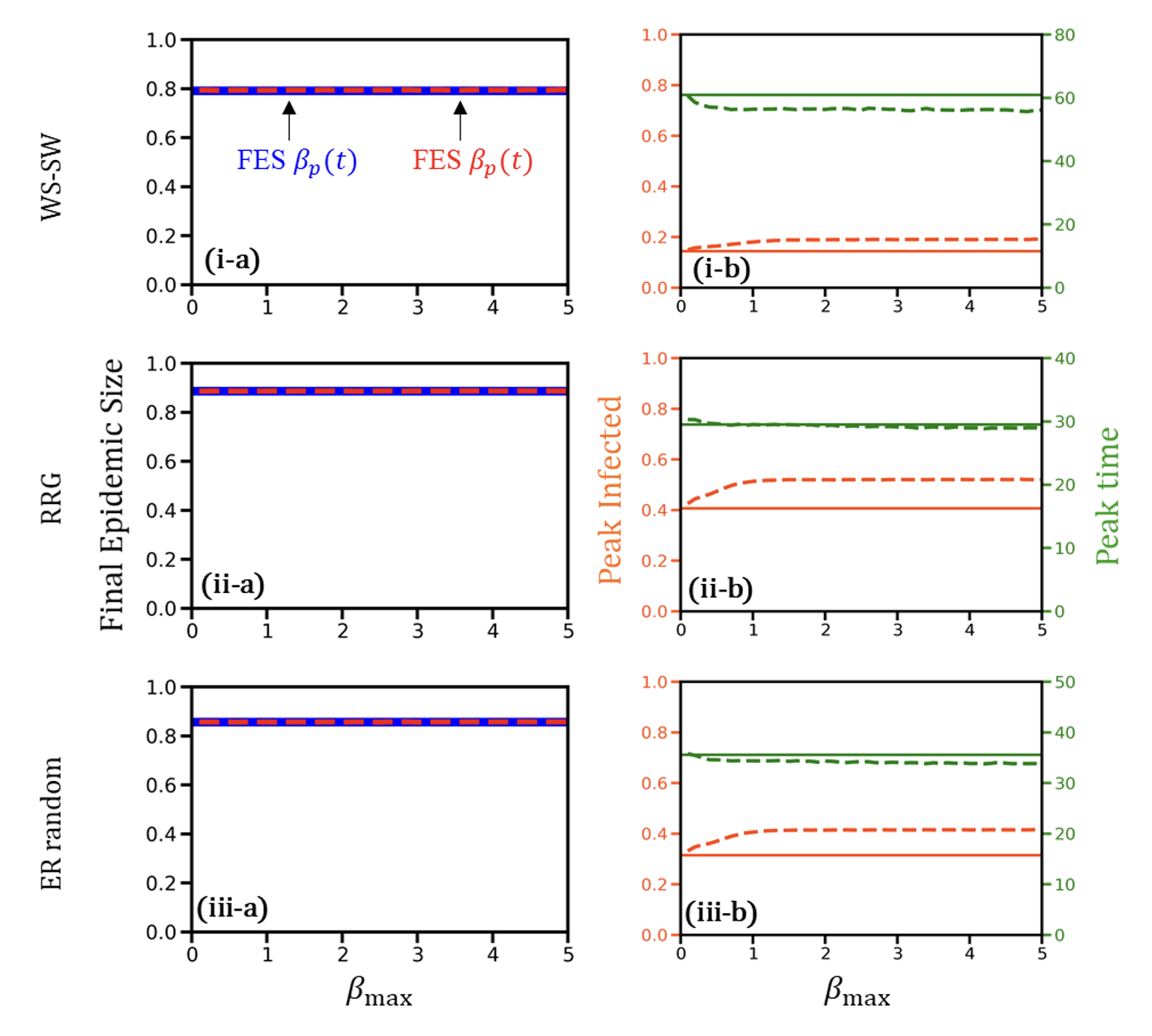

Supplement: S3 Fig — (TIF) [file pone.0295954.s003.tif]

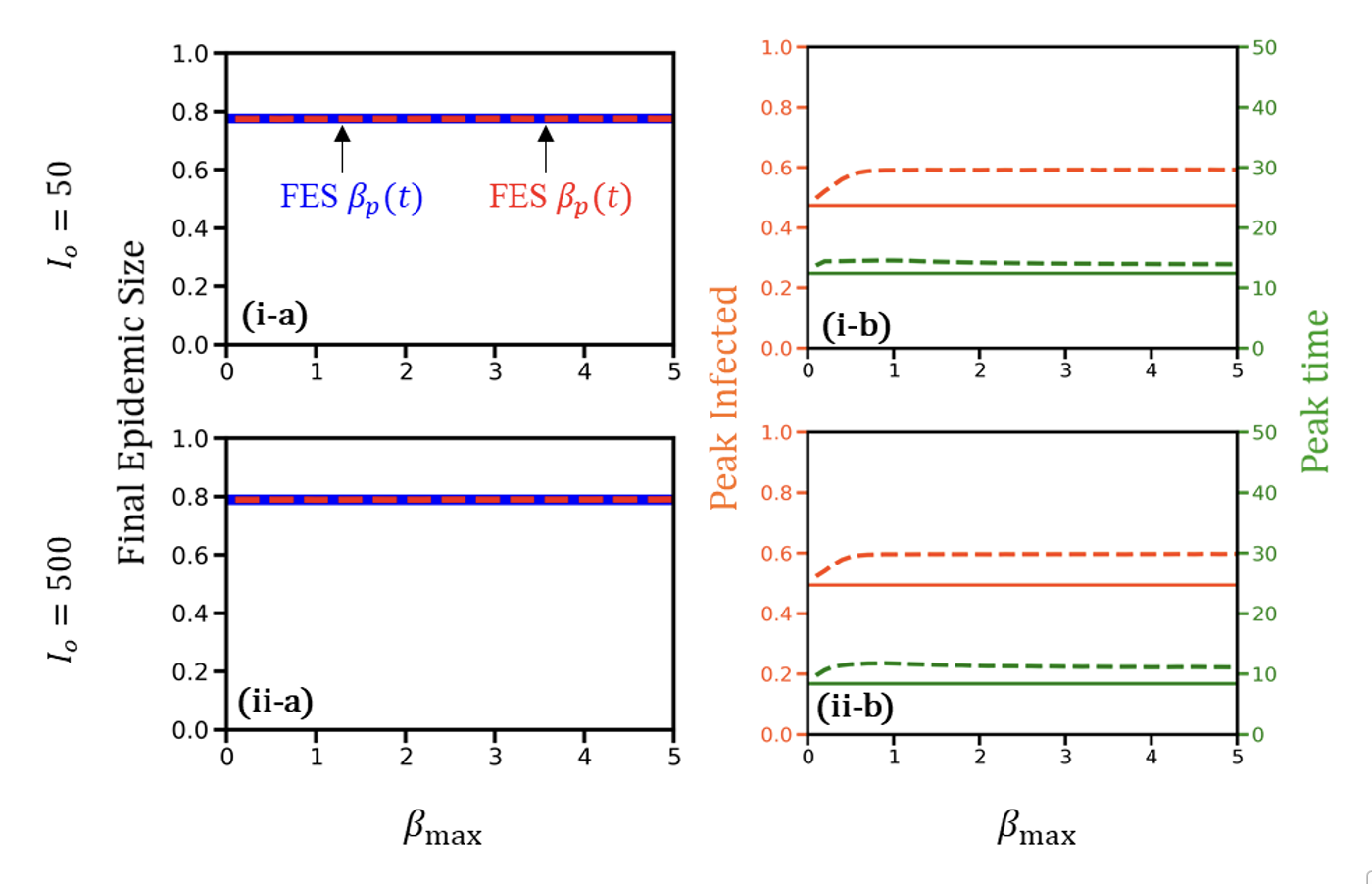

Supplement: S4 Fig — (TIF) [file pone.0295954.s004.tif]

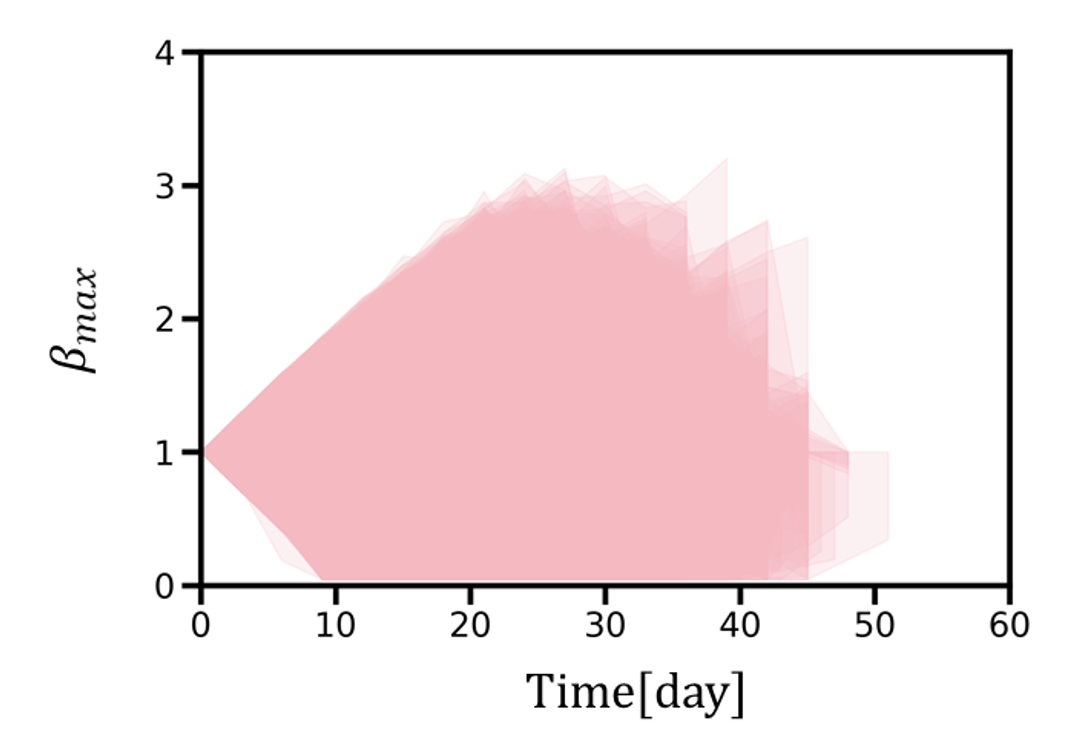

Supplement: S5 Fig — (TIF) [file pone.0295954.s005.tif]
